# Aboveground biomass increments over 26 years (1993–2019) in an old-growth cool-temperate forest in northern Japan

**DOI:** 10.1007/s10265-021-01358-5

**Published:** 2022-01-01

**Authors:** Mahoko Noguchi, Kazuhiko Hoshizaki, Michinari Matsushita, Daiki Sugiura, Tsutomu Yagihashi, Tomoyuki Saitoh, Tomohiro Itabashi, Ohta Kazuhide, Mitsue Shibata, Daisuke Hoshino, Takashi Masaki, Katsuhiro Osumi, Kazunori Takahashi, Wajirou Suzuki

**Affiliations:** 1 Tohoku Research Center, Forestry and Forest Products Research Institute, Morioka, 020-0123 Japan; 2 Department of Biological Environment, Akita Prefectural University, Akita, 010-0195 Japan; 3 Forest Tree Breeding Center, Forestry and Forest Products Research Institute, Hitachi, 319-1301 Japan; 4 Forestry and Forest Products Research Institute, Tsukuba, 305-8687 Japan; 5 Field Science Center, Faculty of Agriculture, Tottori University (Retired), Tottori, 680-8553 Japan; 6 Kansai Research Center, Forestry and Forest Products Research Institute, Kyoto, 612-0855 Japan; 7 Forestry and Forest Products Research Institute (Retired), Tsukuba, 305-8687 Japan

**Keywords:** Forest biomass, Kanumazawa Riparian Research Forest, Long-term data, Temperature

## Abstract

Assessing long-term changes in the biomass of old-growth forests with consideration of climate effects is essential for understanding forest ecosystem functions under a changing climate. Long-term biomass changes are the result of accumulated short-term changes, which can be affected by endogenous processes such as gap filling in small-scale canopy openings. Here, we used 26 years (1993–2019) of repeated tree census data in an old-growth, cool-temperate, mixed deciduous forest that contains three topographic units (riparian, denuded slope, and terrace) in northern Japan to document decadal changes in aboveground biomass (AGB) and their processes in relation to endogenous processes and climatic factors. AGB increased steadily over the 26 years in all topographic units, but different tree species contributed to the increase among the topographic units. AGB gain within each topographic unit exceeded AGB loss via tree mortality in most of the measurement periods despite substantial temporal variation in AGB loss. At the local scale, variations in AGB gain were partially explained by compensating growth of trees around canopy gaps. Climate affected the local-scale AGB gain: the gain was larger in the measurement periods with higher mean air temperature during the current summer but smaller in those with higher mean air temperature during the previous autumn, synchronously in all topographic units. The influences of decadal summer and autumn warming on AGB growth appeared to be counteracting, suggesting that the observed steady AGB increase in KRRF is not fully explained by the warming. Future studies should consider global and regional environmental factors such as elevated CO_2_ concentrations and nitrogen deposition, and include cool-temperate forests with a broader temperature range to improve our understanding on biomass accumulation in this type of forests under climate change.

**Supplementary Information:**

The online version contains supplementary material available at 10.1007/s10265-021-01358-5.

## Introduction

Old-growth forests are widely recognized to play an important role in the carbon cycle (Harmon et al. 1990). It has been commonly accepted that old-growth forests are carbon neutral (Odum 1969) and their living biomass is at ‘steady state’ (Bormann and Likens 1979). However, recent studies indicate that they work as carbon sinks with increasing biomass over centuries (Luyssaert et al. 2008; Tan et al. 2011). Continuous increases in aboveground biomass (AGB) have also been found in temperate (e.g., Foster et al. 2014; Keeton et al. 2011) and tropical (e.g., Baker et al. 2004; Lewis et al. 2009; Phillips et al. 1998; Qie et al. 2017) old-growth forests. Lewis et al. (2004) argued that recent remarkable AGB increases in old-growth Amazonian tropical forests was induced by climate change. On the other hand, in boreal forests biomass growth is more susceptible to climate change in old-growth forests than in young forests, resulting in negative net biomass change (Chen et al. 2016). Thus, assessing climate effects on long-term biomass accumulation in old-growth forests is essential for understanding forest ecosystem functions in a changing climate (McDowell et al. 2020).

Long-term changes in biomass result from the accumulation of short-term changes in the form of gain due to tree growth and loss due to mortality (Hoshizaki et al. 2004). Therefore, to understand how climate affects changes in AGB, the effects of climatic factors on each component need to be taken into account (Chen and Luo 2015; Peña et al. 2018). In addition, endogenous processes such as gap filling in small-scale canopy openings can drive biomass change (McDowell et al. 2020; Phillips et al. 2009): at the local scale, gap formation may cause first a decrease and then an increase in AGB caused by growth promotion of trees around the gap. Repeatedly measured tree census data with tree location can be useful in revealing these processes.

Environmental factors such as topographic position affect both forest biomass (Kubota et al. 2004; Valencia et al. 2009) and tree species composition (Chen and Luo 2015; Kuuluvainen et al. 2017; Ohmann and Spies 1998). For instance, on northern Honshu, Japan, *Fagus crenata* often dominates forest stands on hillslopes, whereas more tree species occur in riparian areas (Suzuki et al. 2002). Tree species in riparian forests have diverse life history traits (e.g., both shorter and longer lifespans, heavy sprouting (Nakamura and Inahara 2007; Sakio 2020)). Therefore, hillslope and riparian stands are expected to differ in the dynamics (i.e., growth and mortality) and, consequently, the pattern of biomass changes in component species. In addition, a recent analysis of long-term tree census data in northern Japan has revealed different responses among species to changing climate and consequent changes in stand structure and species composition (Hiura et al. 2019). Thus, stands with different topographic characteristics can show different responses to climate change.

Here, we quantify decadal changes in AGB and their processes in relation to endogenous processes and climatic factors, using tree census data measured repeatedly over 26 years (1993–2019) in an old-growth, cool-temperate mixed deciduous forest with different types of topographic units in northern Japan. We ask the following questions: (1) Did AGB show net increase or decrease over the whole forest and study period? (2) Did tree species contribute differently to biomass change among the different types of topographic units? (3) How did gain and loss contribute to the overall changes in stand biomass? (4) Did climatic factors and endogenous processes such as canopy gap formation and recovery influence short-term changes in AGB at the local scale?

## Materials and methods

### Study site

**Fig. 1:**
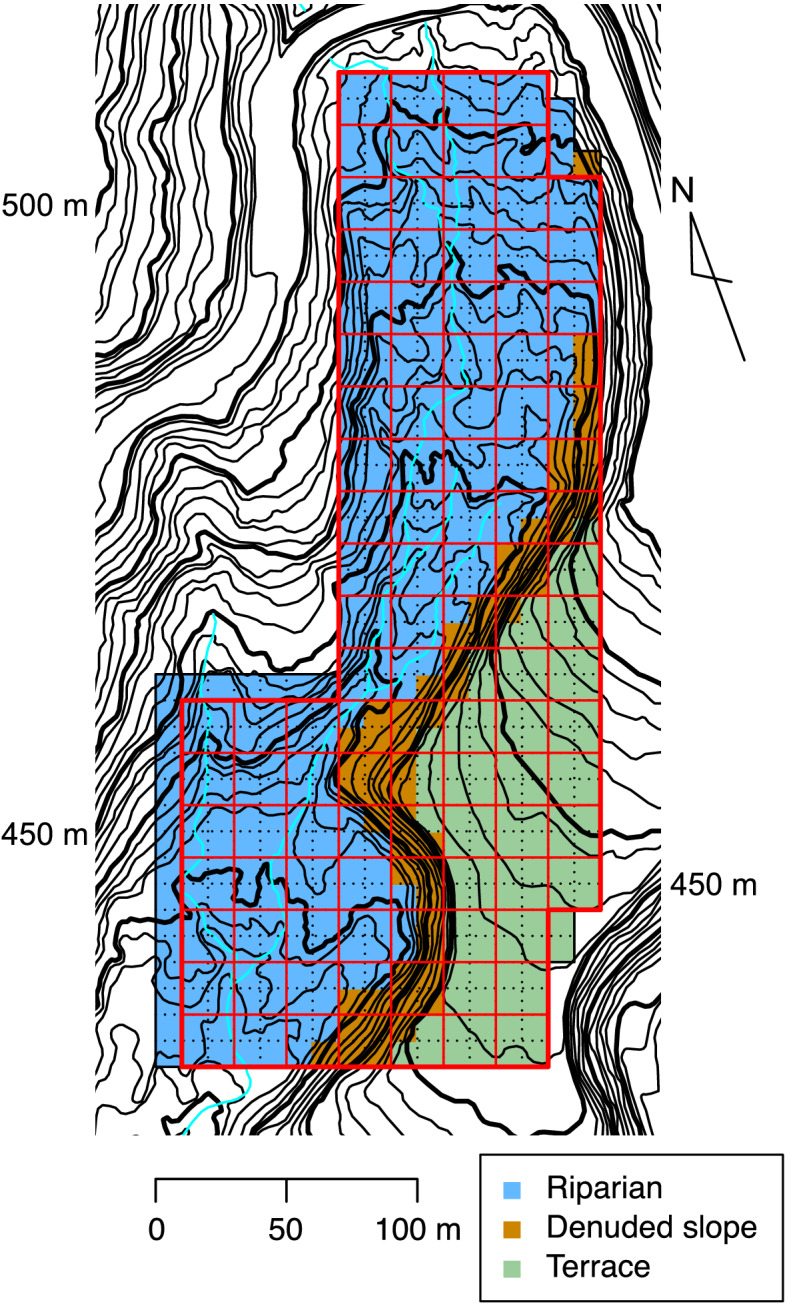
Topographic map of the Kanumazawa Riparian Research Forest (KRRF). The solid frame represents the 4.71-ha KRRF plot. Colors denote the three topographic units: blue, riparian (3.11 ha); orange, denuded slope (0.57 ha); green, terrace (1.03 ha). Black dotted lines show the 10-m × 10-m quadrats; thin red lines show the 20-m × 20-m subplots. Contour interval is 2 m

The study was conducted in the Kanumazawa Riparian Research Forest (KRRF) in Yokodake-Maeyama National Forest, Iwate, northern Japan (39° 06′ 37′′ N, 140° 51′ 17′′ E), an old-growth forest with no record of significant anthropogenic disturbances. In KRRF, tree community dynamics have been repeatedly measured since the establishment of a 4.71-ha permanent plot in 1993 (Fig. 1; Suzuki et al. 2002). This site is one of the core research sites of the Japan Long-Term Ecological Research Network (JaLTER). KRRF has a cool-temperate climate with a mean annual temperature of 8.8 °C and a warmth index (Kira 1991) of 71 °C month (Mahoko Noguchi and Kazuhiko Hoshizaki unpublished data). The mean annual precipitation is 2000 mm, and snow cover lasts 5 months with maximum snow depth of approximately 2 m (Oki et al. 2013). The vegetation depends on the topographic unit. The riparian area is covered with a species-rich deciduous broadleaved forest consisting of both riparian specialists (*Cercidiphyllum japonicum*, *Aesculus turbinata*, *Acer mono*, *Pterocarya rhoifolia*, and *Ulmus laciniata*) and habitat generalists (*Fagus crenata* and *Quercus crispula*) (Masaki et al. 2008; Suzuki et al. 2002). The upper slopes and terrace are dominated by *F. crenata* and *Q. crispula*. Detailed information on the ecology of component species is available in Hoshizaki et al. (1997, 1999), Masaki et al. (2005), and Osumi (2006). The age of the largest *C. japonicum* individual is estimated to be more than 500 years (Osumi 2006), indicating that this forest is sufficiently old-growth. The natural disturbance regime in KRRF is characterized by canopy gap formation and fluvial sediment movements (Oki et al. 2013). Gap-creating disturbance occurs about every 1–3 years, with gap size ranging from tens to hundreds of square meters (Oki et al. 2013). Recent fluvial sediment movements were recorded in 1988, 1998, and 2007, causing ground disturbance with sizes ranging from 144 to 680 m^2^ but no damage to canopy trees.

### Field measurement

The 4.71-ha permanent plot was divided to 471 10-m × 10-m quadrats (Fig. 1). The plot ranges in elevation from 400 to 460 m a.s.l., and includes three topographic units: riparian (3.11 ha), terrace (1.03 ha), and denuded slope between them (0.57 ha). In the whole plot, all stems greater than 5 cm in diameter at breast height (DBH) were tagged for identification and mapped, and DBH was measured at the same marked location on each stem in September or October every 2 years from 1993 to 1999 and every 4 years from then to 2019.

### Estimation of AGB change and its components

We calculated tree AGB, basal area (BA), and stem density in each topographic unit. Individual tree AGB was estimated by using a general allometric equation for tree species in Japan (Ishihara et al. 2015):

1 $$\ln \left( y \right) = - 1.196 + 1.162 \times \ln \left( D \right) + 0.338 \times \left( {\ln \left( D \right))^{2} - 0.044 \times \left( {\ln (D} \right)} \right)^{3} + 0.708 \times \ln \left( \rho \right)$$

where *y* is AGB; *D* is stem DBH, and ρ is the wood specific gravity of each species (Editorial Board of Wood Industry 1966; Hiroko Kurokawa, Masahiro Aiba and Yusuke Onoda unpublished data; Fujiwara et al. 2007). Confidence intervals of changes in AGB, BA, and stem density were estimated via bootstrapping across 10-m × 10-m quadrats following the method of Valencia et al. (2009).

To overview trends in AGB change during the study period and net annual change in AGB, we calculated AGB for three tree size classes: large (≥ 50 cm DBH), medium (15–50 cm DBH), and small (5–15 cm DBH). The net annual change in AGB (in Mg ha^−1^ y^−1^) was calculated each 4-year period from 1996 to 2019 from the tree DBH data of 1995–2019. It was then dissected into annual AGB gain and annual AGB loss. Annual AGB gain was calculated separately for growth of trees in each size class and ingrowth, and AGB loss was calculated for mortality of trees in each size class.

### Analysis of factors affecting short-term AGB gain at local scale

We examined the effects of climatic condition in each measurement period, canopy gap formation and topography on local-scale AGB gain using two linear mixed-effect models (LMMs). For both models, variables were calculated for every 20-m × 20-m subplot in each 4-year period from 1996 to 2019. Subplot size was determined as an appropriate area to detect gap formation and subsequent recovery in consideration of the range of gap size in KRRF. Each 20-m × 20-m subplot was assigned to one of the three topographic units according to the area of the major topographic unit (based on the number of 10-m × 10-m quadrats therein). When the number of 10-m × 10-m quadrats within a 20-m × 20-m subplot were same between two types of topographic units, we referred to the original map of topographic type and identified the area. Both models used AGB gain as the response variable and included subplot as a random effect.

In model 1, we aimed to investigate whether the amount of AGB gain differed among the measurement periods with the effects of topographic unit and gap formation in the current and previous measurement periods. Fixed effects were initial AGB in the current measurement period, AGB losses in the current and previous measurement periods, topographic unit, and the five 4-year measurement periods between 2000 and 2019, with topographic unit and measurement period as categorical variables. Initial AGB was included as it is expected to be the “capital” for AGB gain by tree growth. AGB losses were indices of gap formation in the current and previous measurement periods.

In model 2, the effect of climate was analyzed separately from the effect of measurement period to avoid multicollinearity. Fixed effects in model 2 were initial AGB in the current measurement period, AGB loss in the current and previous measurement periods, topographic unit, and mean air temperature during the previous autumn (September–November) and the current summer (June–August) over the measurement periods. For example, mean air temperature during the previous autumn of the measurement period 1996–1999 is the mean air temperature during September–November, 1995–1998, and that of the current summer is the mean during June–August, 1996–1999.Both types of mean air temperature have a major influence on annual DBH growth of individual trees in most dominant species of KRRF (Michinari Matsushita, Daiki Sugiura and Kazuhiko Hoshizaki manuscript in preparation). As the on-site temperature data do not cover the entire study period, we used data from the nearest weather station, at Wakayanagi (39° 08′ N, 141° 04′ E; 97 m a.s.l.: Japan Meteorological Agency, https://www.data.jma.go.jp/gmd/risk/obsdl/index.php), 18 km east of the study site. In addition to the aforementioned factors, interactions between the topographic unit and climatic factors were included in the model’s fixed effects of the model to examine whether different topographic characteristics shows different responses to climatic factors (model 2.1).

These models were fitted by the lme4 v.1.1-21 package (Bates et al. 2015) in R 3.6.3 (R Core Team 2020). All variables except for categorical variables (i.e., topographic unit and measurement period) were standardized before the analyses. To evaluate the variance explained by the models, we calculated two *R*^2^ values for mixed-effect models following the method of Nakagawa and Schielzeth (2013) and Nakagawa et al. (2017): marginal *R*^2^ (*R*^2^_LMM(m)_), which is the proportion of the variance explained by fixed effects, and conditional *R*^2^ (*R*^2^_LMM(c)_), which is the proportion of the variance explained by both fixed and random effects. These were calculated by the MuMIn v. 1.43.15 package (Bartoń 2020) in R.

## Results

### Overall changes in AGB at plot scale

**Fig. 2:**
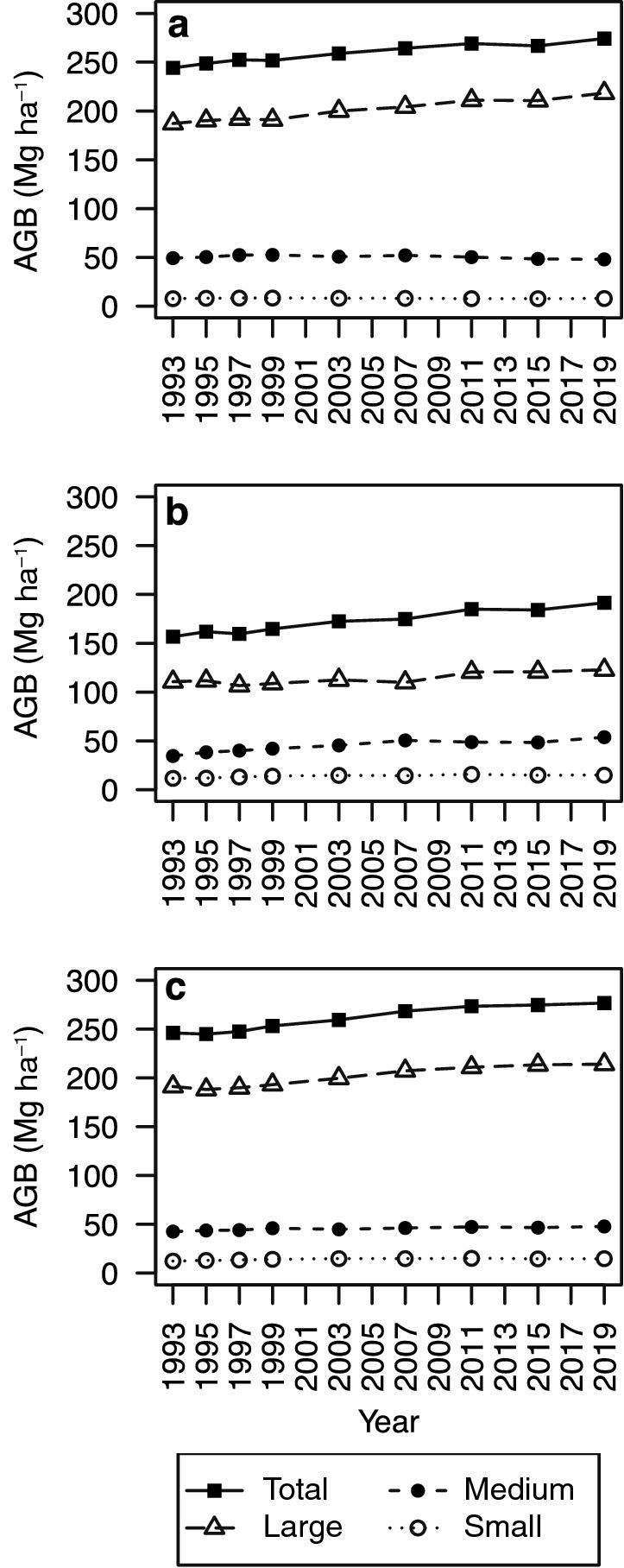
Trends in total aboveground biomass (AGB) over 26 years in the three topographic units. AGB is shown for stand total and stems in three size classes: large (diameter at breast height [DBH] ≥ 50 cm), medium (DBH, 15–50 cm), and small (DBH, 5–15 cm)

BA in 1993 was greatest in the riparian unit (34.2 m^2^ ha^−1^) and least in the denuded slope unit (Table 1). From 1993 to 2019, BA increased significantly in all topographic units. AGB was greatest in the terrace unit (246.0 Mg ha^−1^) at the beginning of the study period (Table 1). It increased significantly in all topographic units during the study period, increasing in most 4-year periods except for some short pauses; for instance, from 2011 to 2015 in the riparian and denuded slope units (Fig. 2). AGB of large trees (≥ 50 cm DBH) in 1993 occupied 76.7% of total AGB in riparian, 70.6% in denuded slope, and 77.7% in terrace units. Trends of increasing total AGB in the riparian and terrace units were similar to those of large-tree AGB. During the study period, stem density declined in the riparian and terrace units but increased in the denuded slope unit (Table 1). The change in stem density was significant only in the riparian unit.

**Table 1:** Basal area, aboveground biomass, and stem density at the study site at the beginning (1993) and end (2019) of the study period with overall changes in three topographic units (riparian, denuded slope, and terrace)

	1993	2019	Change
Basal area (m^2^ ha^−1^)			
Riparian	34.2 (28.7–40.5)	38.6 (32.4–457.7)	4.5 (2.4–6.5)
Denuded slope	21.5 (15.3–28.3)	26.6 (20.2–33.8)	5.1 (2.2–7.8)
Terrace	32.3 (26.6–37.7)	36.6 (30.4–42.6)	4.3 (1.5–6.7)
Aboveground biomass (Mg ha^−1^)			
Riparian	244.1 (202.7–289.4)	274.2 (230.1–326.2)	30.1 (14.0–45.6)
Denuded slope	156.8 (100.3–216.0)	191.5 (136.1–252.7)	34.7 (13.2–56.1)
Terrace	246.0 (202.4–293.5)	276.7 (225.8–336.4)	30.6 (8.0–53.4)
Stem density (stems ha^−1^)			
Riparian	583 (519–648)	509 (452–581)	− 73 (− 111 to − 36)
Denuded slope	781 (637–939)	877 (704–1046)	96 (− 35–235)
Terrace	952 (833–1061)	906 (785–1031)	− 47 (− 118 – 24)

Values in parentheses are 95% confidence intervals. When CIs do not include 0, the changes are significant

In the riparian unit, *C. japonicum* had the largest AGB at the beginning of the study period, followed by *F*. *crenata*, *A*. *turbinata*, *Q*. *crispula*, and *A*. *mono* (Table 2). AGB of these species, except for *Q*. *crispula*, increased during the study period. *Pterocarya rhoifolia* had the greatest increment in AGB over the study period, accounting for 52.3% of the total increment in the riparian unit, followed by *A*. *turbinata* at 25.4%. In contrast, several other species with relatively small AGB at the beginning, such as *Zelkova serrata* and *Ulmus laciniata*, showed a decline in AGB during the study period. The denuded slope and terrace units were dominated by *F*. *crenata* and *Q*. *crispula*, and the denuded slope by *A*. *mono* as well (Table 2). All these species had an increase in AGB during the study period, maintaining the AGB-based rank of species composition.

**Table 2:** Overall changes in aboveground biomass (AGB, in Mg ha^−1^) of component tree species in each topographic unit during the study period and the relative contribution of each species to the total change in AGB. Species are listed in the order of AGB in 1993 in the entire plot

Species	Riparian				Denuded slope				Terrace			
	1993	2019	Change	Contribution (%)	1993	2019	Change	Contribution (%)	1993	2019	Change	Contribution (%)
*Fagus crenata*	52.1	56.2	4.1	13.7	68.9	80.4	11.5	33.2	139.3	153.9	14.7	47.9
*Quercus crispula*	30.6	30.3	− 0.2	− 0.8	30.4	37.8	7.4	21.2	82.1	86.3	4.2	13.7
*Cercidiphyllum japonicum*	58.9	61.9	3.0	10.1	0.0	0.0	0.0	0.0	0.0	0.0	0.0	0.0
*Aesculus turbinata*	47.8	55.5	7.6	25.4	3.8	5.4	1.6	4.5	0.0	0.0	0.0	0.0
*Acer mono*	21.0	21.6	0.5	1.8	28.9	38.0	9.0	26.1	0.2	0.4	0.2	0.6
*Pterocarya rhoifolia*	8.6	24.4	15.8	52.4	0.9	3.7	2.8	8.0	0.0	0.0	0.0	0.0
*Zelkova serrata*	5.7	4.7	− 1.0	− 3.2	2.0	3.6	1.5	4.4	0.0	0.0	0.0	0.0
*Ulmus laciniata*	4.8	2.8	− 2.0	− 6.6	0.0	0.0	0.0	0.0	0.0	0.0	0.0	0.0
*Magnolia obovata*	4.2	6.0	1.8	6.1	0.0	0.5	0.4	1.3	1.2	2.2	1.0	3.3
*Kalopanax pictus*	4.5	5.0	0.5	1.8	0.1	0.7	0.6	1.7	0.0	0.3	0.3	0.9
*Acer sieboldianum*	0.4	0.1	− 0.3	− 1.1	4.5	3.5	− 1.0	− 2.9	6.0	8.0	2.0	6.5
*Acer japonicum*	0.5	0.9	0.4	1.3	1.9	2.4	0.5	1.5	6.0	7.4	1.4	4.5
Others	5.0	4.8	− 0.2	− 0.8	15.3	15.7	0.4	1.1	11.2	18.1	6.9	22.5
Total	244.1	274.2	30.1	100.0	156.8	191.5	34.7	100.0	246.0	276.7	30.6	100.0

**Fig. 3:**
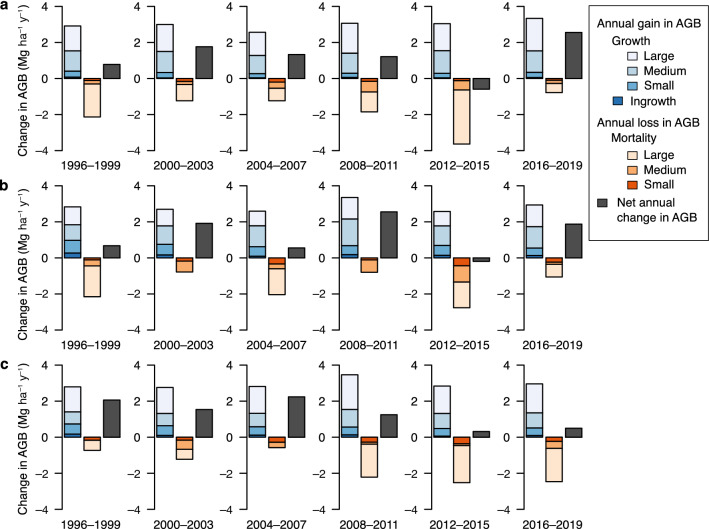
Components of average annual change in aboveground biomass (AGB) by each measurement period in the three topographic units. Blue bars (4 levels of color gradient) denote annual AGB gain from growth of surviving stems in the three size classes (large, diameter at breast height [DBH] ≥ 50 cm; medium, 15–50 cm DBH; small, 5–15 cm DBH) and ingrowth. Orange bars (3 levels of color gradient) denote annual AGB loss from stems that died during each measurement period in the three size classes. Dark gray bars denote net average annual change in AGB

Annual gain in AGB remained at approximately 3 Mg ha^−1^ year^−1^ with some differences among the measurement periods: larger in 2008–2011 and 2016–2019 in all topographic units (Fig. 3). In the riparian and terrace units, large- and medium-sized trees accounted for most of the annual gain. Annual losses in AGB fluctuated among the 4-year periods, and were largest in 2012–2015 in all topographic units. Regardless of topographic unit, measurement periods with greater loss of AGB of large trees tended to have greater total loss of AGB. As a consequence, net annual change in AGB ranged from − 0.6 to + 2.6 Mg ha^−1^ year^−1^ but stayed positive except in 2012–2015 in the riparian and denuded slope units.

### Effects of climate and gap formation on short-term AGB gain at local scale

**Fig. 4:**
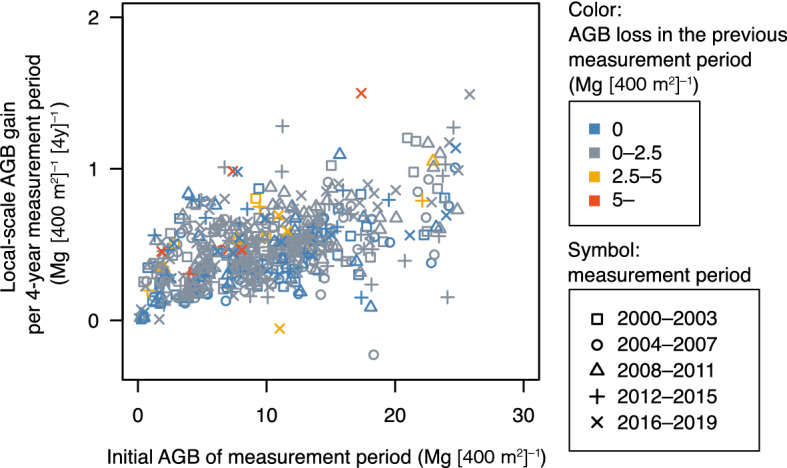
Local-scale aboveground biomass (AGB) gain per 4-year measurement period in relation to initial AGB of measurement period. Colors represent classes of AGB loss in previous measurement period; symbols represent measurement periods

Local-scale AGB gain in the 20-m × 20-m subplots was positively influenced by initial AGB in each measurement period with the largest effect size among the numeric explanatory variables (LMM model 1: Table 3, Fig. 4). It was significantly greater in subplots with larger AGB loss in the previous measurement period but smaller in subplots with larger AGB loss in the current measurement period. It was not significantly affected by topographic unit. It differed significantly among the measurement periods: smaller in 2004–2007 and larger in 2008–2011 and 2016–2019. In model 1, *R*^2^_LMM(m)_ = 0.31 and *R*^2^_LMM(c)_ = 0.76, indicating that 31% of the variation was explained by fixed effects and 76% by fixed and random effects. In model 2 (Table 4), the effects of initial AGB, AGB loss in the current and previous measurement periods, and topographic unit were almost identical to those in model 1. Local-scale AGB gain was larger in measurement periods with higher mean air temperature during the current summer but smaller in those with higher mean air temperature during the previous autumn. The absolute effect size of these two variables was almost equivalent. Model 2 explained almost identical variation as model 1, with *R*^2^_LMM(m)_ = 0.30 and *R*^2^_LMM(c)_ = 0.75. None of the interactions between the topographic unit and climatic factors (i.e., mean air temperature during the previous autumn and the current summer) were significant (model 2.1, Table S2). Additionally, the interactions did not improve performance of the model with *R*^2^_LMM(m)_ = 0.30 and *R*^2^_LMM(c)_ = 0.75 in model 2.1.

**Table 3:** Results of the generalized linear mixed-effect model (model 1) testing the effects of initial aboveground biomass (AGB), canopy gap formation, topographic unit, and measurement period on the AGB gain in 20-m × 20-m subplots

	Estimate	Standard error	df	*t*-value	*P*-value
Initial AGB	0.124	0.015	135.8	8.255	< 0.001
AGB loss by mortality					
Previous	0.016	0.006	511.7	2.819	0.005
Current	− 0.016	0.006	481.0	− 2.782	0.006
Topographic unit (v. Riparian)					
Denuded slope	0.023	0.047	109.3	0.501	0.618
Terrace	− 0.018	0.039	109.0	− 0.461	0.646
Measurement period (v. 2000–2003)					
2004–2007	− 0.050	0.015	442.1	− 3.301	0.001
2008–2011	0.038	0.015	445.1	2.514	0.012
2012–2015	− 0.005	0.015	450.5	− 0.327	0.743
2016–2019	0.040	0.015	455.9	2.606	0.009

All explanatory variables were standardized except for categorical variables (i.e., topographic unit and measurement period)

**Table 4:** Results of the generalized linear mixed-effect model (model 2) testing the effects of initial aboveground biomass (AGB), canopy gap formation, topographic unit, and climate (mean air temperature) of each measurement period on the AGB gain in 20-m × 20-m subplots

	Estimate	Standard error	df	*t*-value	*P*-value
Initial AGB	0.124	0.015	137.9	8.294	< 0.001
AGB loss by mortality					
Previous	0.016	0.006	508.0	2.925	0.004
Current	− 0.014	0.006	481.7	− 2.550	0.011
Topographic unit (v. Riparian)					
Denuded slope	0.023	0.047	109.4	0.498	0.619
Terrace	− 0.018	0.038	109.1	− 0.462	0.645
Mean air temperature					
Previous autumn	− 0.040	0.007	443.0	− 5.865	< 0.001
Current summer	0.040	0.007	450.8	5.803	< 0.001

All explanatory variables were standardized except for categorical variables (i.e., topographic unit)

## Discussion

AGB of KRRF increased steadily over the 26 years in all topographic units, with increments of 30–35 Mg ha^−1^ in each (Table 1). BA also increased over the study period, even though it was initially equivalent to values reported in other cool-temperate old-growth forests in Japan (Masaki et al. 1992; Nakashizuka 1988; Seiwa et al. 2013), indicating that the forest had already been well stocked. These results are consistent with reports that temperate old-growth forests continuously gain biomass over the long term (Keeton et al. 2011; Luyssaert et al. 2008). This continuous stand-scale biomass increment was attributable mainly to an increase in AGB of large trees, in agreement with the reported global importance of large trees in determining stand AGB (Lutz et al. 2018; Slik et al. 2013).

Patterns of tree growth or stand-biomass-change vary across tree species composition and diversity, as well as with environmental conditions such as topography (Kubota et al. 2004; Valencia et al. 2009). In KRRF, topography has been reported to determine tree species distribution through affecting seedling survival and growth differently across species (Masaki et al. 2005). They argued that decreased seedling survival and growth in the terrace unit was caused by lower water availability. However, a steady increase in AGB was common to all three topographic units (Table 1; Fig. 2). Furthermore, neither topographic units (Tables 3 and 4) nor their interaction with climate (Table S2) had distinct influence on local-scale AGB gain. These results suggest that topography-induced differences in water availability do not contribute to spatio-temporal variations in AGB gain of adult trees in KRRF under the recent climate.

We attribute the synchronous increase in AGB among the topographic units with different tree species composition primarily to the AGB increment in *F*. *crenata*, which is dominant in all three topographic units (Table 2). A growing abundance of *F*. *crenata* has been documented in several stable old-growth forests (Seiwa et al. 2013; Yamamoto and Nishimura 1999). Increases in both AGB and stem density of *F. crenata* in KRRF may be due to lack of remarkable disturbance even in the riparian unit during the study period. In the riparian unit, however, the contribution of *F*. *crenata* to the AGB increment is lower than in other topographic units, partially due to the smaller initial dominance of this species. Instead, *Pterocarya rhoifolia*, a riparian specialist of cool-temperate forests in Japan (Sakio et al. 2002), made the largest contribution to the stand AGB increment (Table 2). Despite the substantial decline in its stem density (Table S1), its AGB at the end of the study period was 3 times the initial value. It is likely that the fast growth of *P*. *rhoifolia* (Sakio 1993) is associated with its rapid increase in AGB. Although AGB decreased in some species such as *Z*. *serrata* and *U*. *laciniata* in the riparian unit, *P*. *rhoifolia* compensated for the decrease and resulted in the stand-level AGB increase.

Mortality is the major cause of reduced growth or decline in AGB (Schuster et al. 2008; Xu et al. 2012). Although large disturbances such as strong typhoons, insect outbreaks, or severe flooding in the riparian unit were not recorded during the 26 years, the loss of AGB in KRRF varied substantially among the topographic units and among the 4-year measurement periods (Fig. 3). These variations were explained mainly by the spatio-temporal variation in mortality of large trees. A significant contribution of large-tree mortality to the AGB loss has also been reported in other old-growth forests (Hoshizaki et al. 2004). A large amount of AGB loss in 2012–2015 is due to the mortality of larger-sized trees in this period (Mahoko Noguchi and Kazuhiko Hoshizaki unpublished data). Despite these temporal and spatial variations, the AGB loss generally remained smaller than the AGB gain, bringing about a positive change in AGB in most of the measurement periods. The temporal change of stand-level AGB appears to be inconsistent with the assumed long-term balance between biomass loss caused by canopy gap formation and subsequent gain during gap recovery.

In contrast to AGB loss, temporal fluctuations in AGB gain tended to synchronize across the topographic units at the stand scale (Fig. 3). The results of model 1 indicate that local-scale AGB gain also differed among measurement periods even after adjustment for initial AGB and disturbance during each period (Table 3). As expected, initial AGB positively influenced local-scale AGB gain (Fig. 4). Larger AGB loss in the previous measurement period caused greater AGB gain, suggesting that variations in local-scale AGB gain are partially explained by recovery in and around canopy gaps. Local-scale AGB gain also substantially differed among the measurement periods. The results of model 2 suggest that the observed temporal variations in AGB gain are caused by climatic factors: a warmer current summer had positive effects whereas a warmer previous autumn had negative effects on AGB gain (Table 4). The positive response of tree growth rates to high temperature in the growing season has been reported in deciduous broadleaved species in cool-temperate forests in northern Japan (Hiura et al. 2019). In KRRF, this response is consistent with that of individual-based annual tree growth (Matsushita et al. manuscript in preparation), which often shows considerable inter-annual variations (Ohtsuka et al. 2009) in response to climatic factors (Nabeshima et al. 2010). The negative effect of warmer previous autumn on annual tree growth of deciduous broadleaved species is also found in KRRF (Matsushita et al. manuscript in preparation) and in a cool-temperate forest in central Japan (Shen et al. 2020). Also, over the Northern Hemisphere, autumn warming causes a larger increment in respiration than in photosynthesis and leads to net carbon loss (Piao et al. 2008).

Our models incorporating mean air temperature explained a considerable amount of variation in local AGB gain, although the analysis did not include other potential factors that enhance tree growth such as change in precipitation (Hiura et al. 2019). Both the summer and autumn temperatures at the weather station nearest to KRRF have shown a substantial rise over the past 40 years (Fig. S1). As the positive and negative effects of summer and autumn temperatures were equivalent in absolute size (Table 4), the influences of summer and autumn warming on AGB growth appeared to be counteracting. Therefore, the observed steady AGB increase in KRRF is not fully explained by decadal trends of warming. The simulation results of European temperate forests show that elevated CO_2_ concentrations and nitrogen deposition underpins current high stand growth under the recent climate (Pretzsch et al. 2014). Future studies should consider these global and regional environmental factors, and include cool-temperate forests with a broader temperature range to improve our understanding on biomass accumulation in this type of forests under climate change.

## Supplementary Information

Below is the link to the electronic supplementary material.

Supplementary file1 (PDF 328 KB)

## References

[CR1] Baker TR, Phillips OL, Malhi Y (2004). Increasing biomass in Amazonian forest plots. Philos Trans R Soc Lond B Biol Sci.

[CR2] Bartoń K (2020) MuMIn: Multi-Model Inference. R package version 1.43.17. https://CRAN.R-project.org/package=MuMIn

[CR3] Bates D, Mächler M, Bolker B, Walker S (2015). Fitting linear mixed-effects models using lme4. J Stat Softw.

[CR4] Bormann FH, Likens GE (1979). Pattern and process in a forested ecosystem: disturbance, development, and the steady state based on the Hubbard Brook ecosystem study.

[CR5] Chen HYH, Luo Y (2015). Net aboveground biomass declines of four major forest types with forest ageing and climate change in western Canada’s boreal forests. Global Change Biol.

[CR6] Chen HYH, Luo Y, Reich PB, Searle EB, Biswas SR (2016). Climate change-associated trends in net biomass change are age dependent in western boreal forests of Canada. Ecol Lett.

[CR7] Editorial Board of Wood Industry (1966). Wood materials of Japan.

[CR8] Foster JR, D’Amato AW, Bradford JB (2014). Looking for age-related growth decline in natural forests: unexpected biomass patterns from tree rings and simulated mortality. Oecologia.

[CR9] Fujiwara T, Yamashita K, Kuroda K (2007). Basic densities as a parameter for estimating the amount of carbon removal by forests and their variation. Bull FFPRI.

[CR10] Harmon ME, Ferrell WK, Franklin JF (1990). Effects on carbon storage of conversion of old-growth forests to young forests. Science.

[CR11] Hiura T, Go S, Iijima H (2019). Long-term forest dynamics in response to climate change in northern mixed forests in Japan: a 38-year individual-based approach. For Ecol Manag.

[CR12] Hoshizaki K, Suzuki W, Sasaki S (1997). Impacts of secondary seed dispersal and herbivory on seedling survival in *Aesculus**turbinata*. J Veg Sci.

[CR13] Hoshizaki K, Suzuki W, Nakashizuka T (1999). Evaluation of secondary dispersal in a large-seeded tree *Aesculus**turbinata*: a test of directed dispersal. Plant Ecol.

[CR14] Hoshizaki K, Niiyama K, Kimura K, Yamashita T, Bekku Y, Okuda T, Quah ES, Noor NSM (2004). Temporal and spatial variation of forest biomass in relation to stand dynamics in a mature, lowland tropical rainforest, Malaysia. Ecol Res.

[CR15] Ishihara MI, Utsugi H, Tanouchi H, Aiba M, Kurokawa H, Onoda Y, Nagano M, Umehara T, Ando M, Miyata R, Hiura T (2015). Efficacy of generic allometric equations for estimating biomass: a test in Japanese natural forests. Ecol Appl.

[CR16] Keeton WS, Whitman AA, McGee GC, Goodale CL (2011). Late-successional biomass development in northern Hardwood-Conifer forests of the northeastern United States. For Sci.

[CR17] Kira T (1991). Forest ecosystems of east and southeast Asia in a global perspective. Ecol Res.

[CR18] Kubota Y, Murata H, Kikuzawa K (2004). Effects of topographic heterogeneity on tree species richness and stand dynamics in a subtropical forest in Okinawa Island, southern Japan. J Ecol.

[CR19] Kuuluvainen T, Hofgaard A, Aakala T, Gunnar Jonsson B (2017). North Fennoscandian mountain forests: history, composition, disturbance dynamics and the unpredictable future. For Ecol Manag.

[CR20] Lewis SL, Malhi Y, Phillips OL (2004). Fingerprinting the impacts of global change on tropical forests. Philos Trans R Soc Lond B Biol Sci.

[CR21] Lewis SL, Lopez-Gonzalez G, Sonké B (2009). Increasing carbon storage in intact African tropical forests. Nature.

[CR22] Lutz JA, Furniss TJ, Johnson DJ (2018). Global importance of large-diameter trees. Global Ecol Biogeogr.

[CR23] Luyssaert S, Schulze ED, Börner A, Knohl A, Hessenmöller D, Law BE, Ciais P, Grace J (2008). Old-growth forests as global carbon sinks. Nature.

[CR24] Masaki T, Suzuki W, Niiyama K, Iida S, Tanaka H, Nakashizuka T (1992). Community structure of a species-rich temperate forest, Ogawa Forest Reserve, central Japan. Plant Ecol.

[CR25] Masaki T, Osumi K, Takahashi K, Hozshizaki K (2005). Seedling dynamics of *Acer**mono* and *Fagus**crenata*: an environmental filter limiting their adult distributions. Plant Ecol.

[CR26] Masaki T, Osumi K, Hoshizaki K, Hosino D, Takahashi K, Matsune K, Suzuki W, Sakio H, Tamura T (2008). Diversity of tree species in mountain riparian forest in relation to disturbance-mediated microtopography. Ecology of Riparian Forests in Japan: disturbance, life history, and regeneration.

[CR27] McDowell NG, Allen CD, Anderson-Teixeira K (2020). Pervasive shifts in forest dynamics in a changing world. Science.

[CR28] Nabeshima E, Kubo T, Hiura T (2010). Variation in tree diameter growth in response to the weather conditions and tree size in deciduous broad-leaved trees. For Ecol Manag.

[CR29] Nakagawa S, Schielzeth H (2013). A general and simple method for obtaining R2 from generalized linear mixed-effects models. Methods Ecol Evol.

[CR30] Nakagawa S, Johnson PCD, Schielzeth H (2017). The coefficient of determination R(2) and intra-class correlation coefficient from generalized linear mixed-effects models revisited and expanded. J R Soc Interface.

[CR31] Nakamura F, Inahara S, Johnson EA, Miyanishi K (2007). Fluvial geomorphic disturbances and life history traits of Riparian tree species. Plant disturbance ecology.

[CR32] Nakashizuka T (1988). Regeneration of beech (*Fagus**crenata*) after the simultaneous death of undergrowing dwarf bamboo (*Sasa**kurilensis*). Ecol Res.

[CR33] Odum EP (1969). The strategy of ecosystem development. Science.

[CR34] Ohmann JL, Spies TA (1998). Regional gradient analysis and spatial pattern of woody plant communities of Oregon forests. Ecol Monogr.

[CR35] Ohtsuka T, Saigusa N, Koizumi H (2009). On linking multiyear biometric measurements of tree growth with eddy covariance-based net ecosystem production. Global Change Biol.

[CR36] Oki S, Akiyoshi T, Hoshino D, Shibata M, Matsushita M, Hoshizaki K (2013). Interactive effect of canopy and fluvial disturbances on sapling community structure and species diversity in a montane riparian forest. Ecoscience.

[CR37] Osumi K, Masaki T, Tanaka H, Shibata M (2006). Life history of *Cercidiphyllum**japonicum*: the paradox of early-seral species as a component of climax forest. Forest ecology, with long-term perspectives.

[CR38] Peña MA, Feeley KJ, Duque A (2018). Effects of endogenous and exogenous processes on aboveground biomass stocks and dynamics in Andean forests. Plant Ecol.

[CR39] Phillips OL, Malhi Y, Higuchi N (1998). Changes in the carbon balance of tropical forests: evidence from long-term plots. Science.

[CR40] Phillips OL, Higuchi N, Vieira S, Baker TR, Chao K-J, Lewis SL (2009). Changes in Amazonian forest biomass, dynamics, and composition, 1980–2002. Amazon Global Change.

[CR41] Piao S, Ciais P, Friedlingstein P (2008). Net carbon dioxide losses of northern ecosystems in response to autumn warming. Nature.

[CR42] Pretzsch H, Biber P, Schütze G, Uhl E, Rötzer T (2014). Forest stand growth dynamics in Central Europe have accelerated since 1870. Nat Commun.

[CR43] Qie L, Lewis SL, Sullivan MJP (2017). Long-term carbon sink in Borneo’s forests halted by drought and vulnerable to edge effects. Nat Commun.

[CR44] R Core Team (2020). R: a language and environment for statistical computing.

[CR45] Sakio H (2020). Long-term ecosystem changes in Riparian Forests.

[CR46] Sakio H, Kubo M, Shimano K, Ohno K (2002). Coexistence of three canopy tree species in a riparian forest in the Chichibu Mountains, Central Japan. Folia Geobot.

[CR47] Sakio H (1993). Sapling growth patterns in *Fraxinus* and *Pterocarya**rhoifolia*. Jpn J Ecol.

[CR48] Schuster WSF, Griffin KL, Roth H, Turnbull MH, Whitehead D, Tissue DT (2008). Changes in composition, structure and aboveground biomass over seventy-six years (1930–2006) in the Black Rock Forest, Hudson Highlands, southeastern New York State. Tree Physiol.

[CR49] Seiwa K, Miwa Y, Akasaka S (2013). Landslide-facilitated species diversity in a beech-dominant forest. Ecol Res.

[CR50] Shen Y, Fukatsu E, Muraoka H, Saitoh TM, Hirano Y, Koh Y (2020). Climate responses of ring widths and radial growth phenology of *Betula**ermanii*, *Fagus**crenata* and *Quercus**crispula* in a cool temperate forest in central Japan. Trees.

[CR51] Slik JWF, Paoli G, McGuire K (2013). Large trees drive forest aboveground biomass variation in moist lowland forests across the tropics. Global Ecol Biogeogr.

[CR52] Suzuki W, Osumi K, Masaki T, Takahashi K, Daimaru H, Hoshizaki K (2002). Disturbance regimes and community structures of a riparian and an adjacent terrace stand in the Kanumazawa Riparian Research Forest, northern Japan. For Ecol Manag.

[CR53] Tan Z-H, Zhang Y-P, Schaefer D, Yu G-R, Liang N, Song Q-H (2011). An old-growth subtropical Asian evergreen forest as a large carbon sink. Atmos Environ.

[CR54] Valencia R, Condit R, Muller-Landau HC, Hernandez C, Navarrete H (2009). Dissecting biomass dynamics in a large Amazonian forest plot. J Trop Ecol.

[CR55] Xu C-Y, Turnbull MH, Tissue DT, Lewis JD, Carson R, Schuster WSF, Whitehead D, Walcroft AS, Li J, Griffin KL (2012). Age-related decline of stand biomass accumulation is primarily due to mortality and not to reduction in NPP associated with individual tree physiology, tree growth or stand structure in a *Quercus*-dominated forest. J Ecol.

[CR56] Yamamoto S, Nishimura N (1999). Canopy gap formation and replacement pattern of major tree species among developmental stages of beech (*Fagus**crenata*) stands, Japan. Plant Ecol.

