# Supplementary material for: Aboveground biomass increments over 26 years (1993–2019) in an old-growth cool-temperate forest in northern Japan
Source: J Plant Res. 2022 Jan 1;135(1):69–79. doi: 10.1007/s10265-021-01358-5 (PMC8755688; doi:10.1007/s10265-021-01358-5)
Supplement: Supplementary file 1 — Supplementary file1 (PDF 328 KB) [file 10265_2021_1358_MOESM1_ESM.pdf]

Electronic supplementary materials

Title:

Aboveground biomass increments over 26 years (1993–2019) in an old-growth cool-temperate forest in northern Japan

Authors:

Mahoko Noguchi, Kazuhiko Hoshizaki, Michinari Matsushita, Daiki Sugiura, Tsutomu Yagihashi, Tomoyuki Saitoh, Tomohiro Itabashi, Ohta Kazuhide, Mitsue Shibata, Daisuke Hoshino, Takashi Masaki, Katsuhiro Osumi, Kazunori Takahashi, Wajirou Suzuki

Journal:

Journal of Plant Research

Corresponding author:

Mahoko Noguchi

Tohoku Research Center, Forestry and Forest Products Research Institute

92-25 Nabeyashiki, Shimokuriyagawa, Morioka, Iwate 020-0123, Japan

Tel: +81-19-648-3941

Fax: +81-19-641-6747

E-mail: mahoko@ffpri.affrc.go.jp

Content:

Tables S1–S2

Fig. S1

Table S1 Overall changes in stem density (ha^{-1}) of component tree species in each topographic unit during the study period. Species are listed in the same order as in Table 2 (i.e., the order of aboveground biomass [AGB] in 1993 in the entire plot).

Species	Riparian			Denuded slope			Terrace		
	1993	2019	Change	1993	2019	Change	1993	2019	Change
Fagus crenata	68	90	22	107	146	39	87	91	4
Quercus crispula	15	11	−4	44	46	2	43	41	−2
Cercidiphyllum japonicum	86	82	−4	0	0	0	0	0	0
Aesculus turbinata	42	39	−3	4	5	2	0	0	0
Acer mono	36	39	3	68	60	−9	1	1	0
Pterocarya rhoifolia	101	62	−39	46	23	−23	0	0	0
Zelkova serrata	13	10	−2	5	5	0	0	0	0
Ulmus laciniata	40	24	−16	2	2	0	0	0	0
Magnolia obovata	13	13	0	2	4	2	6	6	0
Kalopanax pictus	6	1	−5	4	4	0	1	1	0
Acer sieboldianum	2	3	1	30	40	11	30	39	9
Acer japonicum	23	31	8	46	75	30	181	200	19
Others	136	103	−33	425	468	44	604	527	−77
Total	583	509	−73	781	877	96	952	906	−47

Table S2 Results of the generalized linear mixed-effect model (model 2.1) testing the effects of initial aboveground biomass (AGB), canopy gap formation, topographic unit, climate (mean air temperature) of each measurement period, and interactions between topographic unit and climate on the AGB gain in 20-m × 20-m subplots.

	Estimate	Standard error	df	*t*-value	*P*-value
Initial AGB	0.123	0.015	137.9	8.262	<0.001
AGB loss by mortality					
Previous	0.016	0.006	508.0	2.919	0.004
Current	−0.014	0.006	481.4	−2.533	0.012
Topographic unit (v. Riparian)					
Denuded slope	0.023	0.047	109.3	0.494	0.622
Terrace	−0.018	0.038	109.0	−0.462	0.645
Mean air temperature					
Previous autumn	−0.047	0.008	442.9	−5.893	<0.001
Current summer	0.041	0.008	450.8	5.830	<0.001
Interactions between topographic unit and mean air temperature					
Denuded slope: previous autumn	0.019	0.020	441.1	0.954	0.340
Terrace: previous autumn	0.020	0.017	441.0	1.173	0.242
Denuded slope: current summer	−0.002	0.020	441.0	−0.090	0.928
Terrace: current summer	−0.002	0.017	441.0	−0.125	0.900

All explanatory variables were standardized except for categorical variables (i.e., topographic unit).

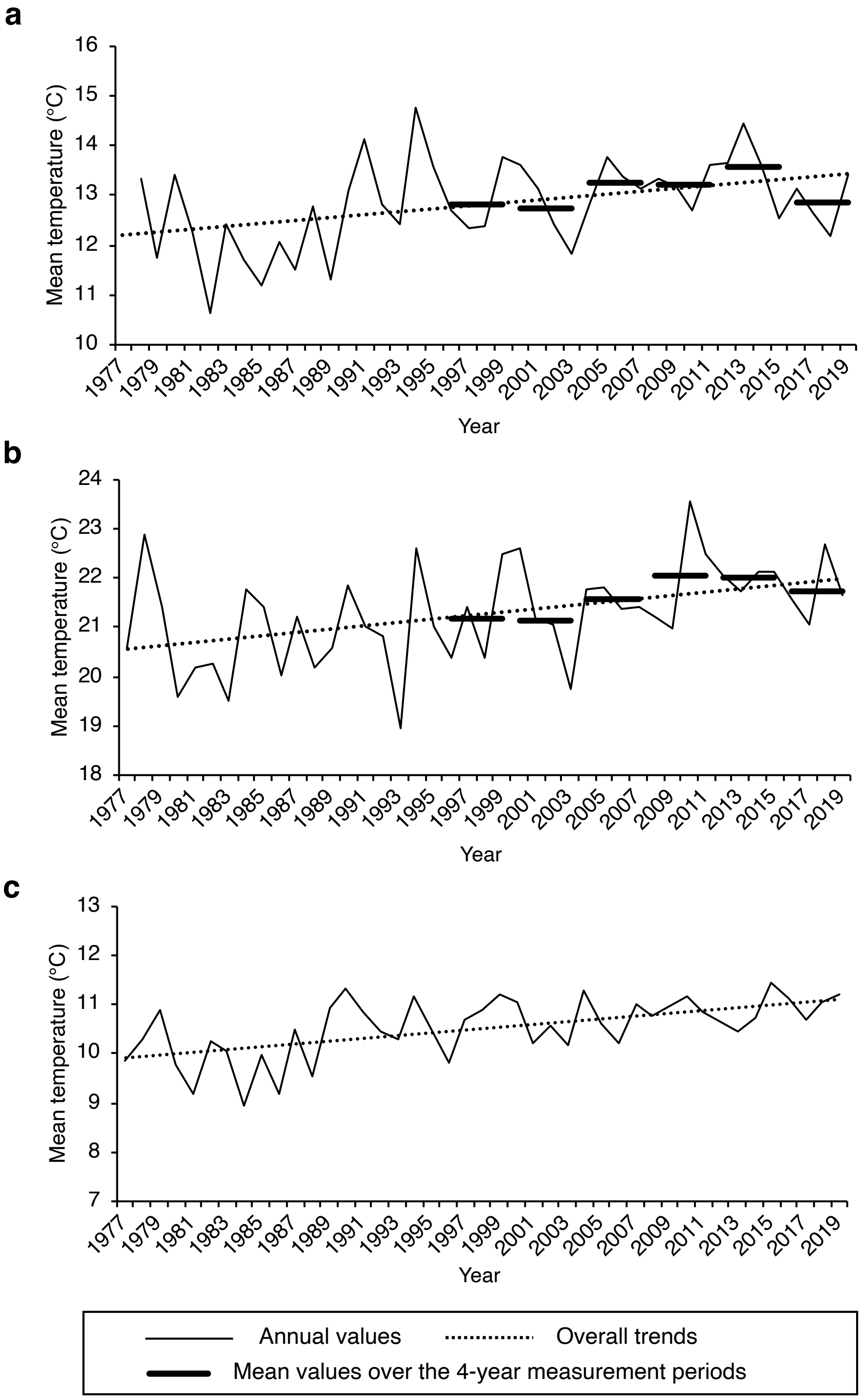

Fig. S1 Trends in mean air temperature of (a) the previous autumn and (b) the current summer, and (c) annual mean air temperature at the nearest weather station, Wakayanagi (39°08′N, 141°04′E; 97 m a.s.l.: Japan Meteorological Agency, https://www.data.jma.go.jp/gmd/risk/obsdl/index.php). Solid lines denote annual values; dotted lines denote overall trends. Solid horizontal bars denote the mean values over the six 4-year measurement periods (only shown for a and b).
